# The effects of laser displacement on femtosecond laser-assisted conjunctival autograft preparation for pterygium surgery

**DOI:** 10.1371/journal.pone.0245223

**Published:** 2021-01-14

**Authors:** Valencia Hui Xian Foo, Yu-Chi Liu, Hon Shing Ong, Marcus Ang, Jodhbir S. Mehta

**Affiliations:** 1 Singapore National Eye Centre, Singapore, Singapore; 2 Singapore Eye Research Institute, Singapore, Singapore; 3 Ophthalmology and Visual Science Academic Clinical Research Program, Duke-National University of Singapore Graduate Medical School, Singapore, Singapore; Bascom Palmer Eye Institute, UNITED STATES

## Abstract

**Aims:**

To evaluate the effects of no-suction femtosecond laser (FSL) stability on conjunctival autograft (CAG) dissection in pterygium surgery.

**Methods:**

Prospective analysis of 35 eyes from 34 subjects who underwent femtosecond laser-assisted pterygium surgery with the Ziemer Z8 laser (Ophthalmic Systems AG, Switzerland). Intraoperative absolute FSL displacements were measured and correlated with the duration and ease of CAG peel, CAG thickness, measured with intraoperative optical coherence tomography, and deviation from intended graft thickness.

**Results:**

The median absolute FSL displacement was 22 μm (interquartile range [IQR] 14.7 to 60.8), while median vertical FSL displacement was 14.7 μm (IQR 7.3 to 44) and median horizontal FSL displacement was 22.0 μm (IQR 14.7 to 44). 65.7% had a grade 1 peel, 11.4% had grade 2 peel, 14.3% had grade 3 peel and 8.6% had grade 4 peel. The median duration of CAG peel was 5.4 seconds (IQR 3 to 21.4). The median CAG thickness was 69 μm (IQR 60.3 to 78.5), and the median deviation from targeted graft thickness was 9 μm (IQR 1 to 16). Eyes with more difficult peels and longer duration of CAG peels had significantly greater vertical FSL displacements (p = 0.04 and 0.02 respectively), but not horizontal displacement, age, ethnicity, CAG thickness or deviation from original thickness, compared to those with better quality and shorter duration peels. 1 eye (2.9%) had an incomplete CAG peel with a buttonhole and 2 eyes had graft tears (5.7%).

**Conclusion:**

Micro-displacements during the suction-free CAG preparation are common but they did not affect the quality of the CAG peel, duration of peel, or CAG thickness. However, vertical globe displacement during FSL-assisted CAG creation was significantly associated with a more difficult and longer CAG peel duration. This highlights the importance of the cornea traction suture fixation to ensure stability of the eye during FSL application.

## Introduction

Pterygium is a common ocular surface disease characterised by fibrovascular growth arising from the conjunctiva and extending onto the cornea [1]. It is more common in peri-equatorial latitudes due to elevated levels of ultraviolet radiation [2], with prevalence rates ranging from 2.8% to 33% [3, 4]. Surgical removal may be indicated when there is: reduction in visual acuity from induced astigmatism or encroachment onto the visual axis, cosmetic deformity, or ocular inflammation unresponsive to medical therapy [5]. While there are various surgical techniques available, the lowest recurrence rates have been observed when pterygium excisions are performed with conjunctival autografts (CAG) [6]. However, one of the main challenges faced by surgeons in this technique is the ability to create a uniformly thin graft that is free of Tenon’s capsule, to reduce the risks of graft retraction and pterygium recurrence [7].

Femtosecond lasers (FSL) are infrared lasers with a wavelength of 1053 nm and an ultrashort pulse duration (10^−15^) [8]. Their capacity to perform precise geometric cuts of different patterns via photo-disruption while preserving surrounding tissues allows for their applications in refractive, corneal, and cataract surgeries [9, 10]. Currently, there are 9 commercially available FSL devices, each with differing energy impulse emission and frequency [11, 12]. The LDV Z8 (Ziemer Ophthalmic Systems AG, Port, Switzerland) has a larger numerical aperture compared to other systems; hence it can emit energy in the nanojoule range, with high repetition rates above 1 megahertz, enabling it to cut through translucent tissue like the conjunctiva [13]. Translating this favourable characteristic of FSL into pterygium surgery, we have successfully incorporated the LDV Z8 to enhance the efficacy of CAG dissection in a procedure called femtosecond laser-assisted pterygium surgery (FLAPS) [14, 15]. Animal studies found high reproducibility in the creation of ultrathin CAGs (designated at 60 μm) regardless of surgeon experience [14]. Early clinical trials have shown good cosmesis and minimal scarring at the bulbar surface and graft site in human eyes [15, 16]. Additionally, anterior segment optical coherence tomography angiography imaging suggested rapid revascularisation of both host and donor beds [17]. However, the theoretical advantage of potentially reusing the CAG harvest site would still need to be evaluated in large studies, such as in glaucoma filtration surgeries, since a large area of superior conjunctiva is harvested in FLAPS.

Despite the promising clinical outcomes, there are a few points that have to be considered. Firstly, the LDV Z8 is programmed to harvest a standardised ellipsoid CAG of 7 mm x 10 mm diameter (55.0 mm^2^) using the lamellar keratoplasty module. Even though these dimensions could be made smaller, it cannot presently be enlarged beyond this size. However, in our initial clinical trial, we have not found the need for a larger graft [16]. Secondly, the FSL machine’s articulating handpiece allows the surgeon to applanate evenly on the superior bulbar conjunctival surface. During the laser application, there is no suction applied throughout this step. In all other uses of the FSL in cornea, refractive and cataract surgery, suction is applied to ensure ocular stability [18]. To minimise movement, the eye is secured with a superior corneal fixation suture. However, micro-movements can occur during the laser application resulting in uneven photo-disruption which may give rise to an uneven dissection of the CAG. The resultant CAG could be more adhesive to peeling resulting in graft tears. Subsequent inadequate coverage of the host scleral bed might lead to a higher risk of pterygium recurrence.

To better understand the consequences of FSL displacements on the resultant CAG, we evaluated intraoperative displacements during FSL-assisted CAG dissection and their associations with a) total duration taken and ease of CAG peel (primary outcome), and b) resultant CAG thickness and deviation of graft thickness from the programmed cutting depth of 60 μm (secondary outcome)

## Methods

### Recruitment of patients and surgical technique

This prospective cohort study was carried out from January 2016 to June 2019. We recruited 35 eyes of 34 consecutive patients with pterygium from the Singapore National Eye Centre, including 1 patient with a double-headed pterygium. The research adhered to the tenets of the Declaration of Helsinki and ethics committee approval was obtained from the Institutional Review Board of Singhealth (IRB number: 2016/2512). Written informed consent was obtained from all subjects.

**Fig 1:**
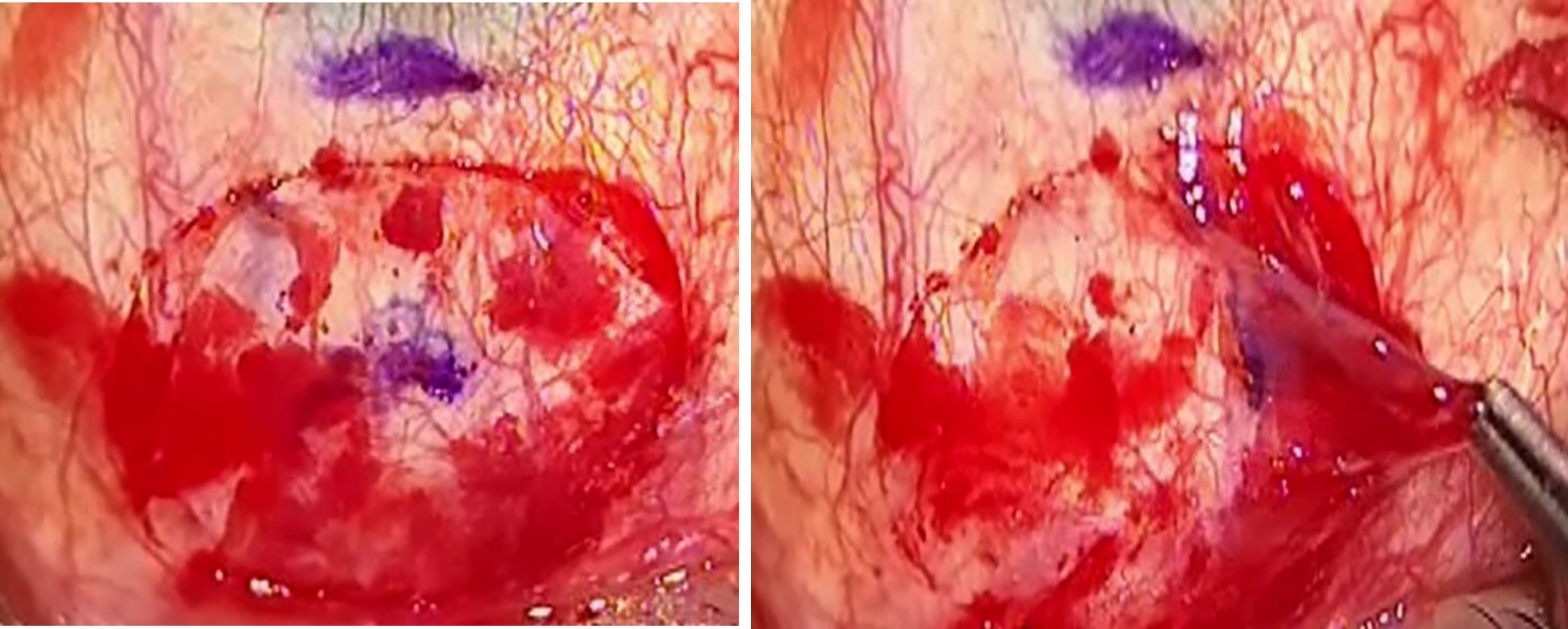
**(A)** Outline of CAG after FSL dissection. **(B)** Lift and peeling of CAG with conjunctival forceps.

The surgery was performed by 2 corneal surgeons (J.M and M.A) using a previously described technique [15]. In brief, under peribulbar anaesthesia, a 7–0 vicryl corneal traction suture (Ethicon, Somerville, MA) was placed in the peripheral superior cornea to control the eye position and the body of the pterygium was excised at the level of the limbus. The head was removed from the corneal surface with a pterygium Mini-Blade (Beaver-Visitec, Waltham, MA). The remaining pterygium and subconjunctival Tenon’s tissue was removed to expose bare sclera. The Ziemer LDV Z8 was programmed to harvest an ellipsoid CAG of 7 mm (vertical) x 10 mm (horizontal) (55.0mm^2^), and 60 μm depth on the superior bulbar conjunctiva, using the lamellar keratoplasty module. The superior bulbar conjunctiva was marked with a surgical skin marker approximately 4 mm from the limbus (Secureline; Aspen Surgical, Caledonia, MI) and the Z8 handpiece was applanated onto the bulbar conjunctival surface. Centration was performed by the surgeon during applanation, ensuring that the conjunctival marking was in the middle of the crosshair on the Z8 visualization screen. Once adequate centration was achieved, the ocular surface was gently applanated with the laser head, with the tear meniscus displaced beyond the programmed resection area representing good and even applanation. Throughout the creation of the CAG, the Z8 laser head must be held steady for a total of 22 seconds. Immediately after laser cutting, central CAG thickness was measured by the in-built optical coherence tomography (OCT) at the graft centre and 2 mm from the centre on either side. The CAG was peeled (**Fig 1**), positioned, and glued onto the area of the conjunctival defect with fibrin glue (Artiss; Baxter, CA). A bandage contact lens was then applied. The postoperative regimen consisted of topical preservative-free dexamethasone 0.1% and levofloxacin 0.5% every three hours for 1 week, followed by a tapering dose over 8 weeks.

### Measurement of displacements throughout femtosecond laser application, duration and grading of peel of conjunctival autograft

**Fig 2:**
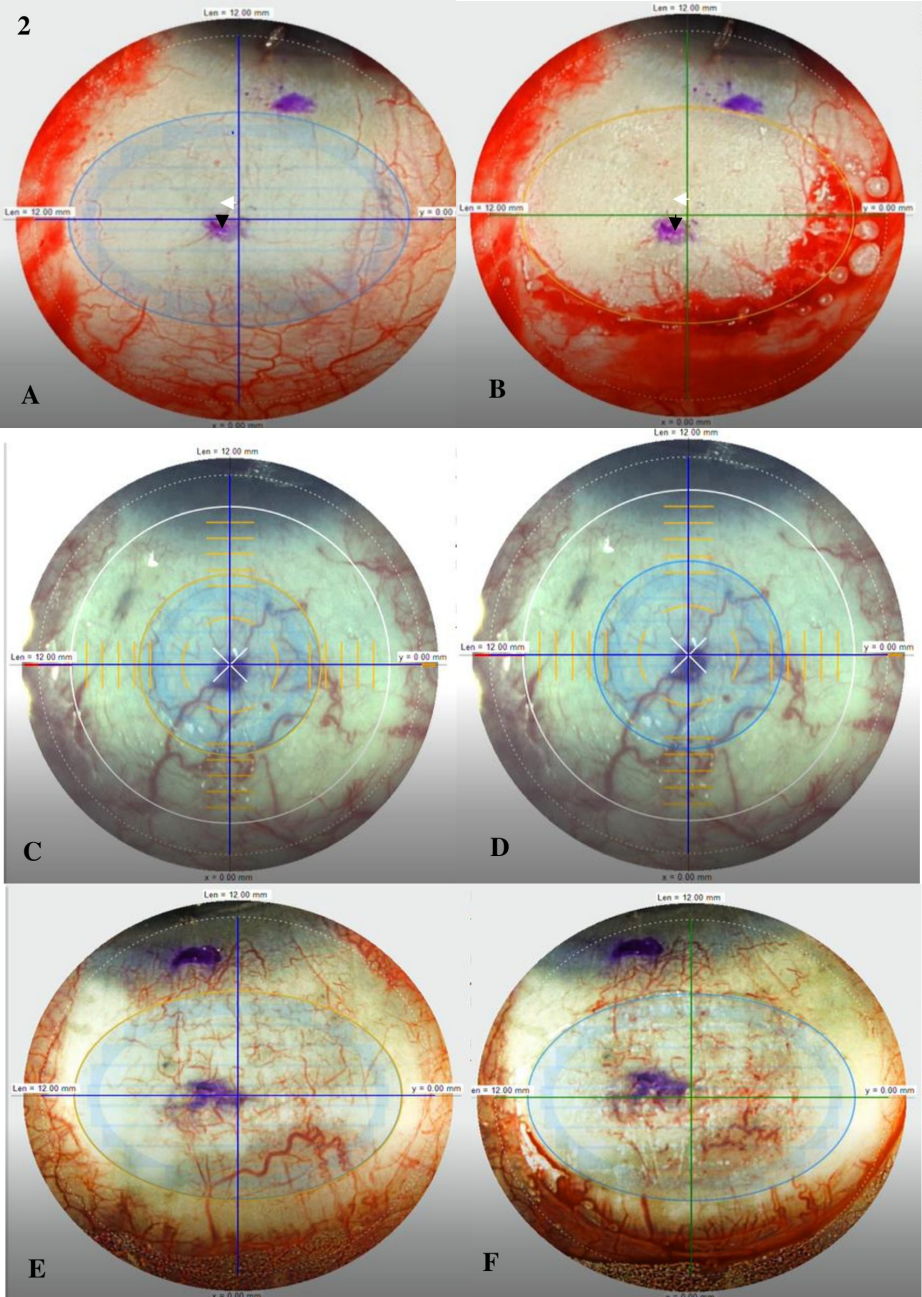
**(A)** Before FSL dissection. **(B)** After FSL dissection. **White arrow**: horizontal distance from crosshair to centre of conjunctival ink marking. **Black arrow:** vertical distance from crosshair to centre of conjunctival ink marking. Horizontal displacement is the difference between the measurements of the 2 white arrows, and vertical displacement is the difference between the measurements of the 2 black arrows. Absolute displacement distance is calculated by taking the square root of the total sum of the individual squares of the horizontal and vertical displacements. Minimal horizontal and vertical displacement noted before. **(C)** Before FSL dissection. **(D)** After FSL dissection. This eye showed minimal horizontal and vertical displacements. **(E)** Before FSL dissection. **(D)** After FSL dissection. A greater amount of horizontal and vertical displacements are noted in this eye, compared to **(C)** and **(D)**.

Video recordings of the FLAPS cases were saved and evaluated by a single masked grader (V.F). Two specific video frames of one pre- and one post-laser application were used for measurements. The CAG dissection has a horizontal lamellar cut (duration of 10 seconds) that takes place before the vertical rim cut (duration of 9 seconds). The horizontal and vertical distances from the pre- and post-laser frames were measured in microns from the centre of the conjunctiva marking to the centre of the crosshair on the Z8 visualisation screen using ImageJ software. Deviations were calculated by taking the absolute differences between the pre- and post-laser measurements. To calculate absolute displacement distances, the square root of the combined sum of the individual squares of the horizontal and vertical displacements was calculated. (**Fig 2A and 2B**).

Based on observations of the surgical videos, a single masked grader (V.F) measured the time taken in seconds for the surgeon to peel the CAG from the conjunctival bed from start to end based on the surgical videos. Measurements of duration were repeated three times; the average duration of the three measurements was used for analyses. The ease of CAG peel was graded on the following scale–Grade 1: easy peel with no adhesion to the surface, Grade 2: easy peel with minimal adhesion to surface, but a complete separation of CAG from the surface, Grade 3: moderate resistance to peel but a complete separation of CAG from the surface, and Grade 4: resistance to peel requiring manual dissection of CAG.

### Statistical analysis

A sample size of 35 was calculated to produce a two-sided 95% confidence interval with a width equal to 0.300 (a hypothesized and estimated correlation coefficient between absolute FSL displacement and our primary outcome of CAG peel and grade, as this was a pilot study) when the estimate of Spearman’s rank correlation of 0.80. All statistical analyses were performed using Statistical Package for the Social Sciences version 22.0 (SPSS, Inc, Chicago, IL). Values were expressed as median (interquartile range [IQR] 25–75 percentile). An intragroup comparison was performed using the Wilcoxon signed-rank test for continuous variables, intergroup comparison performed with the Kruskal-Wallis test for continuous variables, and McNemar Chi-square test for categorical variables. Correlation analyses were carried out using Spearman coefficient for non-parametric variables. p < 0.05 was considered statistically significant.

## Results

There were 35 eyes of 34 subjects. Half were male. The median age of subjects was 65 (IQR 55.5–72) years old. The majority of subjects (74.3%) were Chinese, 2 (5.71%) were Malay, 2 (5.71%) Indian and 4 (14.3%) (2 from Asian continents and 2 from Western continents) were of other races.

Median absolute FSL displacement was 22 μm (IQR 14.7 to 60.8, range 0 to 526), while median vertical FSL displacement was 14.7 μm (IQR 7.3 to 44, range 0 to 403) and median horizontal FSL displacement was 22.0 μm (IQR 14.7 to 44, range 0 to 389). Majority of eyes (n = 23, 65.7%) had a grade 1 peel, 4 (11.4%) had grade 2 peel, 5 (14.3%) had a grade 3 peel, and 3 (8.6%) had a grade 4 peel. The median duration of CAG peel was 5.4 seconds (IQR 3 to 21.4). The median central 4 mm CAG thickness was 69 μm (IQR 60.3 to 78.5 μm) and the median deviation from targeted CAG thickness being 9 μm (IQR 1 to 16). (**Table 1**).

**Table 1:** Baseline characteristics of participants according to the absolute displacement of FSL laser during CAG dissection.

		Median (IQR) / n (%)		
	All N = 35	Displacement ≤100 μm N = 31^#^	Displacement >100 μm N = 4	p*
Age, years	65 (55.5–72)	65 (58.5–72.2)	60.5 (41.8–65)	0.22
Ethnicity				0.35
Chinese	26 (74.3)	24 (77.4)	2 (50)	
Malay	2 (5.7)	1 (3.2)	1 (25)	
Indian	2 (5.7)	2 (6.5)	0	
Others	5 (14.3)	4 (12.9)	1 (25.0)	
Gender, male	17 (48.7)	15 (48.4)	2 (50.5)	0.99
Timing of Peel, s	5.4 (3–21.4) (15.43)	5 (3–21.4)	6.0 (3.6–47.5)	0.69
Grading of Peel				0.39
1	23 (65.7)	20 (64.5)	3 (75)	
2	4 (11.4)	3 (9.7)	1 (25)	
3	5 (14.3)	5 (16.1)	0	
4	3 (8.6)	3 (9.7)	0	
Graft Thickness, μm	69 (60.3–78.5)	67.5 (60–80)	72.0 (70–72)	0.48
Deviation from Original Graft Thickness, μm	9 (1–16)	8.0 (1–18.5)	11.0 (8–11)	0.99

Data are expressed as numbers (percentages) for categorical variables or median (interquartile range [IQR]) for continuous variables

*Based on McNemar chi-square or Wilcoxon signed-rank test, comparing characteristics between participants of different FSL displacements

# 2 eyes had no measured displacements

Median vertical displacements were found to be significantly higher in eyes with a worse grading of peel compared to those with a better grading of peel (Grade 1 = 14.7 μm [IQR 0 to 22], Grade 2 = 33.0 μm [IQR 29.3 to 53.2], Grade 3 = 44.0 μm [IQR 7.3 to 235], Grade 4 = 102.7 μm [IQR 7.3 to 102.7], p = 0.04), but horizontal displacements were not significantly different amongst eyes of different grades of peels (Grade 1 = 14.7 μm [IQR 7.3 to 44.0], Grade 2 = 18.3 μm [IQR 3.7 to 110.0], Grade 3 = 29.3 μm [IQR 18.3 to 150.3], Grade 4 = 22 μm [IQR 14.7 to 22.0], p = 0.86). Eyes with worse grades of peel also had a significantly longer duration of CAG peel (p<0.001) (**Table 2**). We did not find significant differences in age, gender, ethnicity, central 4mm graft thickness, or deviation from original planned graft thickness amongst eyes with various grades of CAG peel (p > 0.05 for all) (**Table 2**).

**Table 2:** Comparison of eyes with better versus. Worse grade of CAG peel.

			Median (IQR) / n (%)		
	Grade 1 N = 23	Grade 2 N = 4	Grade 3 N = 5	Grade 4 N = 3	p*
Age, years	65 (58.5–72.3)	59.5 (44.8–69.8)	70 (44–76.9)	68 (61–68)	0.85
Ethnicity					0.81
Chinese	17 (73.9)	3 (75)	4 (80)	2 (50)	
Malay	2 (8.9)	0	0	0	
Indian	1 (4.4)	0	0	1 (25)	
Others	3 (13)	1 (25)	1 (20)	0	
Gender, male	11 (47.8)	3 (75)	1 (20)	0	0.37
Absolute Displacements of FSL, μm	16.4 (10.4–30.2)	31.1 (17.5–214.5)	23.2 (18.6–58.7)	67.6 (16.6–71.1)	0.94
Horizontal Displacements of FSL, μm	14.6 (7.3–44)	18.3 (3.7–110)	29.3 (18.3–150.3)	22 (14.7–22)	0.86
Vertical Displacements of FSL, μm	14.7 (0–22)	33 (29.3–53.2)	44 (7.3–235)	102.7 (7.3–102.7)	**0.04**
Timing of Peel, s	4 (3–5.4)	17.1 (14.3 0 50.6)	27 (23–35.5)	24 (17.2–24)	**< 0.001**
Graft Thickness, μm	69 (60.8–78.5)	79 (79–79)	71 (58–71)	61.5 (60–61.5)	0.66
Deviation from Original Graft Thickness, μm	9 (1.5–15)	31 (31–31)	1 (-2–1)	12 (0–12)	0.30

Data are expressed as numbers (percentages) for categorical variables or median (interquartile range [IQR]) for continuous variables

*Based on One way Anova test or McNemar Chi-square test, comparing characteristics between participants of different grades of CAG peel

We further grouped the study cohort according to the duration of CAG peel (Group 1 = 0 to 3s, Group 2 = 3.1 to 5.4s, Group 3 = 5.5 to 21.4s and Group 4 = 21.5s and longer). Median vertical displacements were also found to be significantly higher in eyes with a longer CAG peel time (Group 1 = 7.3 μm [IQR 0 to 18.3], Group 2 = 14.7 μm [IQR 3.7 to 18.3], Group 3 = 29.3 μm [IQR 14.7 to 33.0], Group 4 = 62.3 μm [IQR 22.0 to 125.0], p = 0.02), but horizontal displacements were not significantly different amongst eyes with different CAG peel duration (Group 1 = 14.7 μm [IQR 3.7 to 22.0], Group 2 = 22.0 μm [IQR 11.0 to 40.3], Group 3 = 22.0 μm [IQR 14.7 to 59.0], Group 4 = 70.0 μm [IQR 16.5 to 139.3], p = 0.28). We did not find significant differences in age, gender, ethnicity, grade of peel, graft thickness or deviation from original planned central 4 mm graft thickness amongst eyes with different duration of peel (p > 0.05 for all).

We examined the study cohort according to absolute displacement, with the first group consisting of eyes with an absolute displacement of ≤ 100μm (n = 31), and the second consisting of eyes with an absolute displacement of >100μm (n = 4). Between these 2 groups, we did not find significant differences in terms of age, gender, ethnicity distribution, duration of peel, grading of ease of peel, resultant central 4 mm CAG thickness, or deviation from the originally planned graft thickness between groups (**Table 1**).

Between the 2 corneal surgeons, we did not find significant differences in terms of absolute, horizontal or vertical FSL displacement, ease, and duration of peel or central CAG thickness and deviation of central 4mm CAG thickness. However, 1 surgeon had a higher median of CAG peel duration than the other (16.1s, IQR 6.4–32.3 vs 5s, IQR 3–20.3, p = 0.16). 1 out of 35 eyes (2.9%) had a complicated CAG peel with a buttonhole that led to an incomplete peel. 2 out of 35 eyes (5.7%) had peripheral graft tears (with 1 graft having both buttonhole formation with a peripheral tear of the CAG). However, all of these grafts could be used satisfactorily.

**Fig 3:**
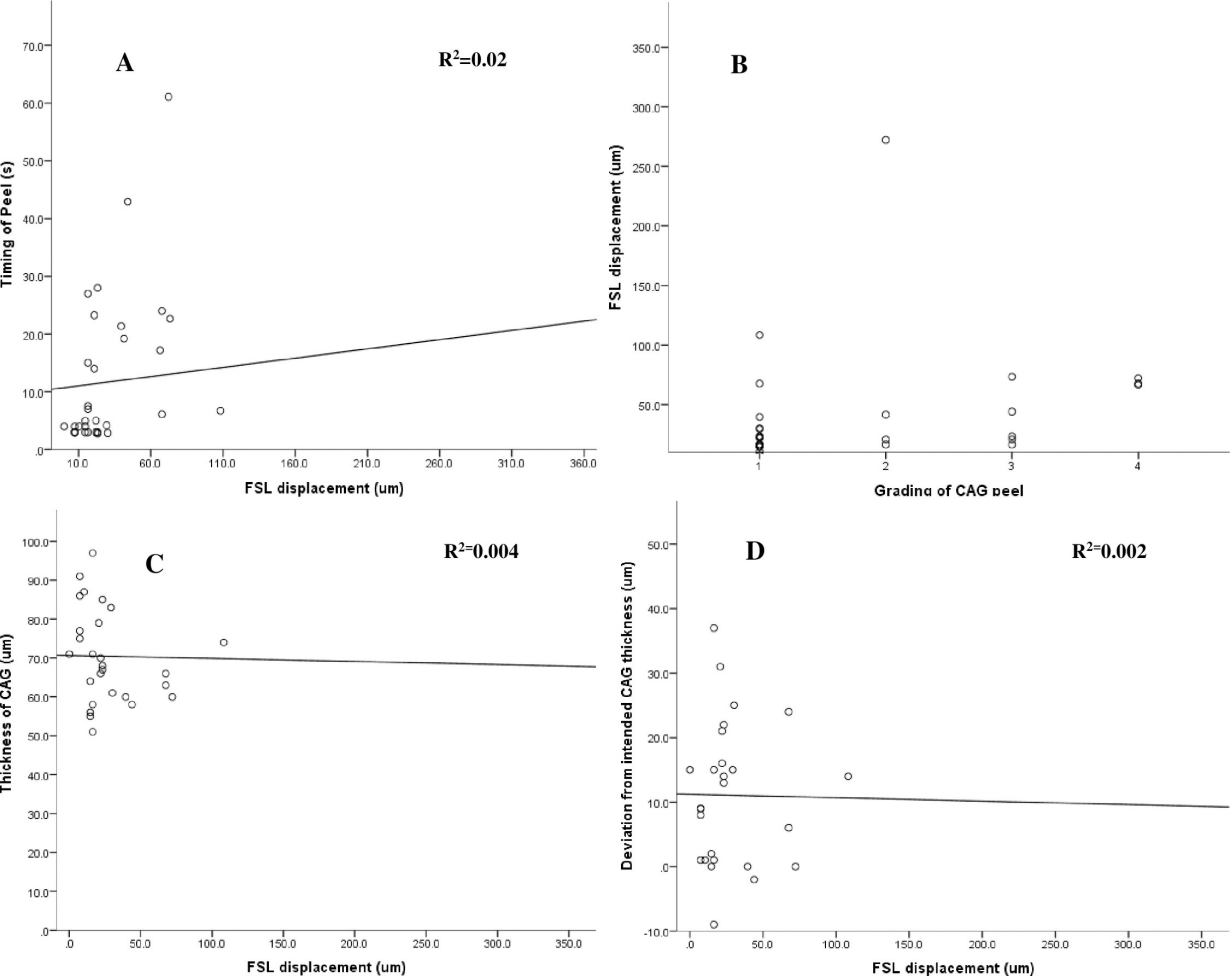
Scatterplots showing relationships between absolute displacement of FSL (μm) [range: 0–525.8 μm–outlier removed from graph] and different parameters such as with: **(A)** Duration of peel (s) [range: 2.8–61.08s] (R^2^ = 0.02, p = 0.002). **(B)** Grading of peel (p = 0.03). **(C)** CAG thickness [range: 51 to 97 μm] (R^2^ = 0.004, p = 0.7), and **(D)** Deviation from intended CAG thickness [range: -9 to 37 μm] (R^2^ = 0.002, p = 0.7).

**Fig 4:**
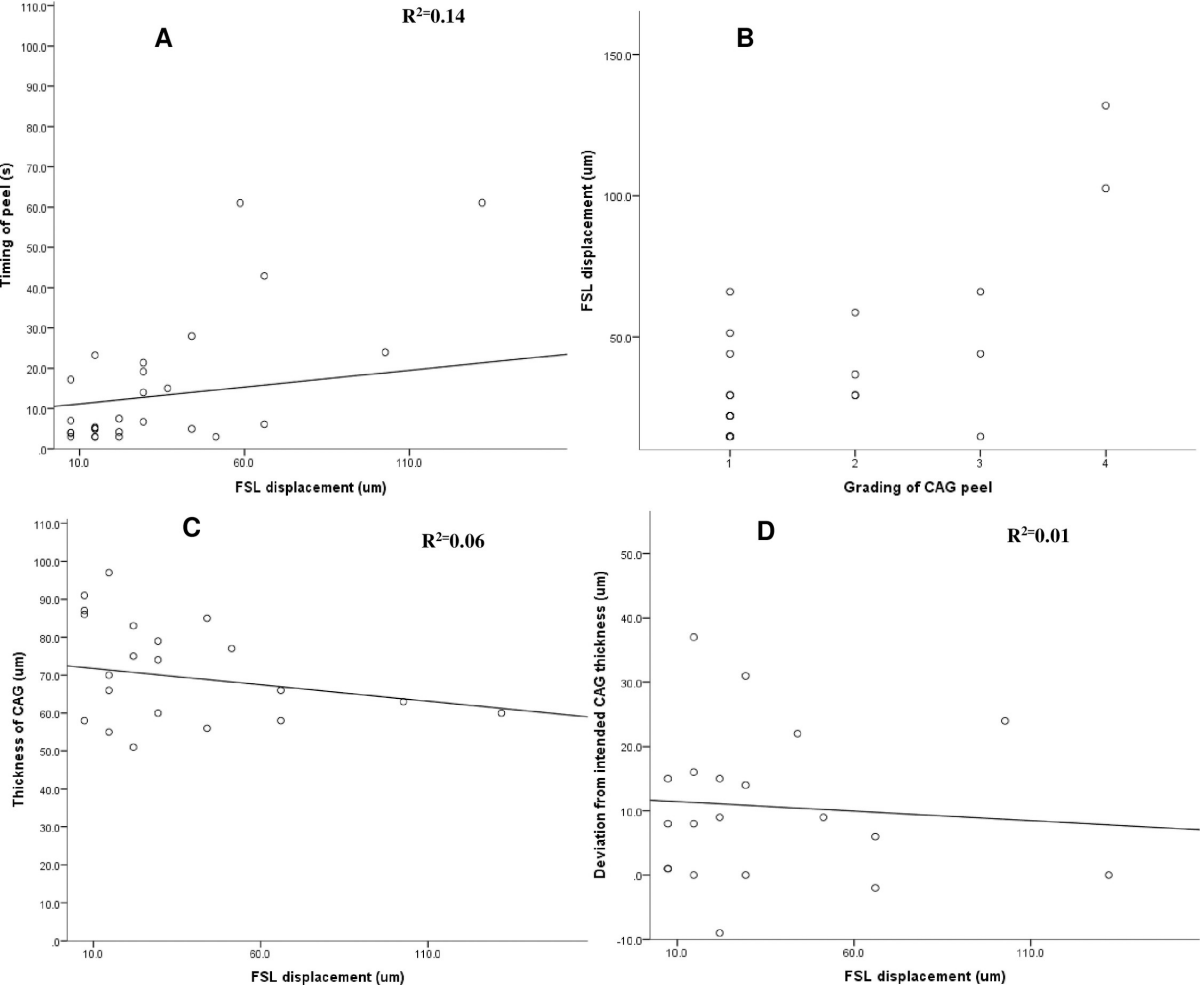
Scatterplots showing relationships between vertical displacement of FSL (μm) [range: 0–403 μm–outlier removed from graph] and different parameters such as with: **(A)** Duration of peel (s) [range: 2.8–61.08s] (R^2^ = 0.14, p <0.001). **(B)** Grading of peel (p = 0.01). **(C)** CAG thickness [range: 51 to 97 μm] (R^2^ = 0.06, p = 0.4). **(D)** Deviation from intended CAG thickness [range: -9 to 37 μm] (R^2^ = 0.01, p = 0.5).

**Fig 5:**
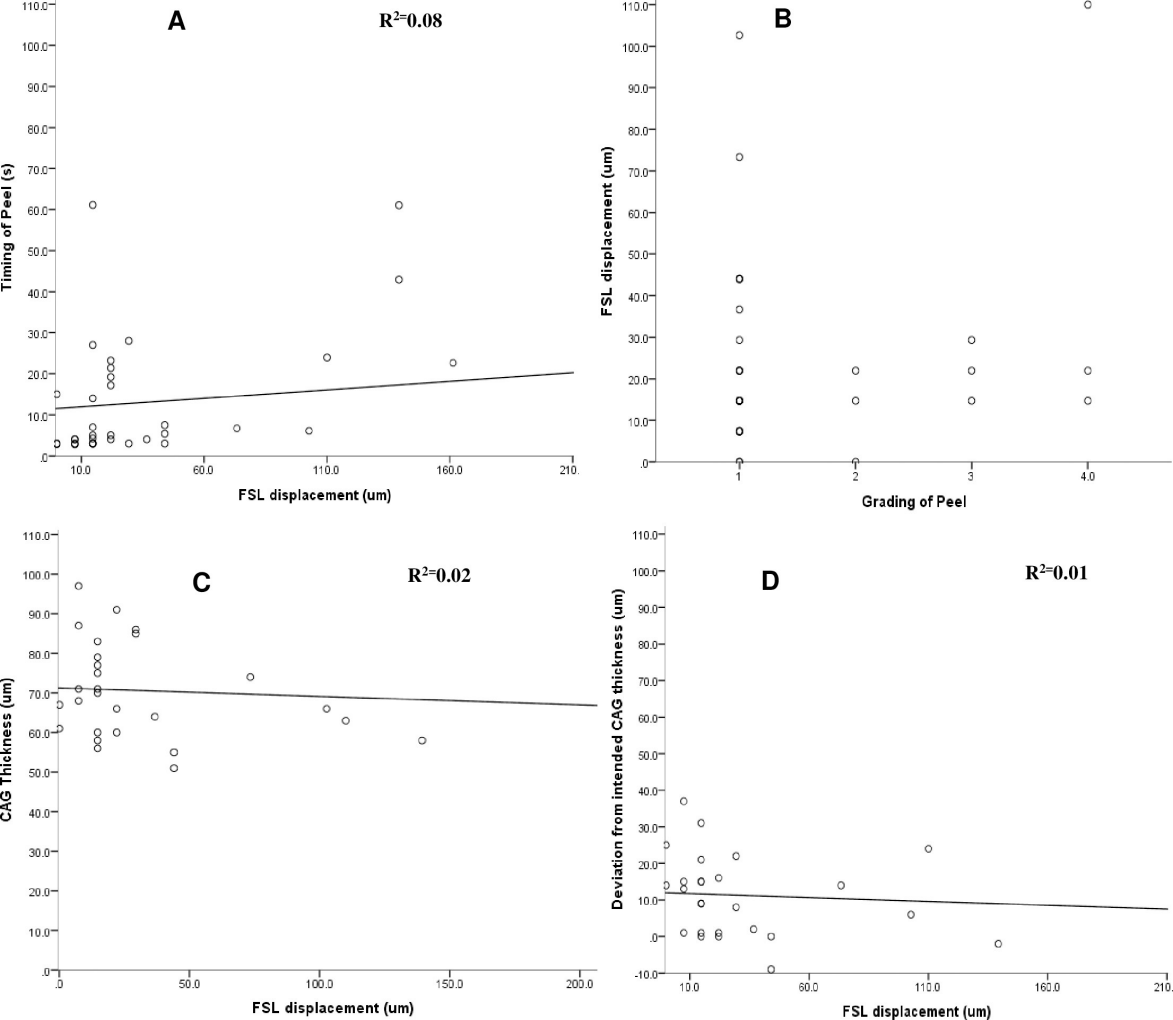
Scatterplots showing relationships between horizontal displacement of FSL (μm) [range: 0–388.7 μm–outlier removed from graph] and different parameters such as with: **(A)** Duration of peel (s) [range: 2.8–61.08s] (R^2^ = 0.08, p = 0.01). **(B)** Grading of peel (p = 0.2). **(C)** CAG thickness [range: 51 to 97 μm] (R^2^ = 0.02, p = 0.2), and **(D)** Deviation from intended CAG thickness [range: -9 to 37 μm] (R^2^ = 0.01, p = 0.06).

We plotted the relationships between absolute (**Fig 3**), vertical (**Fig 4**), and horizontal (**Fig 5**) FSL displacements with the grade of CAG peel, duration of peel, CAG thickness, and deviation of planned CAG thickness respectively. Vertical FSL displacement showed a weak linear and positive correlation (R^2^ = 0.14) with the grading of the duration of peel (**Fig 4A)**. The relationship between absolute, vertical, and horizontal FSL displacements with other CAG parameters did not show significant linear relationships (R^2^< 0.1 for all).

## Discussion

Our study found that vertical FSL displacements during suction-free CAG dissection was associated with lower quality and longer duration of CAG peel, but did not affect the final CAG thickness. However, horizontal or absolute FSL displacements were not associated with the quality or duration of CAG peel or thickness. This study highlights the importance of ensuring vertical stability of the globe during CAG dissection, such as with a secured and externally fixed corneal traction suture that stabilises the downward rotation of the eye. Overall, the consistency and efficiency of FSL grafts dissection remain largely uncompromised despite the presence of absolute micro-displacements.

CAG dissection with the FSL laser comprises of 2 parts; the horizontal lamellar cut following by the vertical rim cut. In our study, the median vertical FSL displacement was 14.7 μm, the median horizontal FSL displacement was 22.0 μm and the median absolute FSL displacement was 22.0 μm. We found that the eyes with significantly greater vertical FSL displacements, but not absolute or horizontal, had a worse grade and longer duration of CAG peel. Although there are multiple statistical comparisons made amongst groups that might increase a chance of a false positive result, the reported p-value here likely represents a significant and biological plausible trend towards a poorer grade of CAG peel with greater vertical FSL displacement, and hence a correction for multiple comparisons was not made. CAG dimensions are standardised at 7mm (vertical) x 10mm (horizontal). We feel that displacements in the shorter vertical axis resulted in a greater impact in terms of peel quality or duration over horizontal and absolute displacements. At the superior bulbar conjunctiva surface, the natural curvature of the globe changes more in the vertical than the horizontal axis, leading to a more uneven applanation of the FSL in the vertical than the horizontal axis. Due to the loose compactness of the conjunctival stromal tissue, and the larger diameter of the horizontal cut, there is a greater chance that the cavitation bubbles will spread horizontally than vertically. Furthermore, during the vertical rim cut, the traction suture that might have loosened towards the end of the FSL application could contribute to vertical displacement. The fixation suture must therefore be secured well, keeping the eye retracted inferiorly throughout the whole laser procedure, especially in patients with a strong Bell’s reflex. Horizontal displacement can be controlled by the surgeon much more easily than vertical displacement, with the surgeon keeping his elbows supported on the headrest during laser head applanation.

Micro-movements are inevitable in the no-suction FSL assisted CAG harvest, with just 2 out of 35 eyes (5.7%) in our study having no measured displacements. These movements could cause the FSL to dissect through different planes of tissue, possibly resulting in an incomplete CAG cut and resistance to CAG peeling. Despite the presence of vertical FSL displacements which were significantly associated with lower quality and longer duration of CAG peel, we reported an overall short median duration of CAG peel of 5.35 seconds, with the majority of peel durations lasting between 3 to 21.37s. Only 2 cases had a CAG peel duration longer than 60 seconds. A Vannas scissors was used in these 2 cases to complete the manual dissection and CAG peel to prevent the propagation of the CAG tear or buttonhole during peeling.

**Fig 6:**
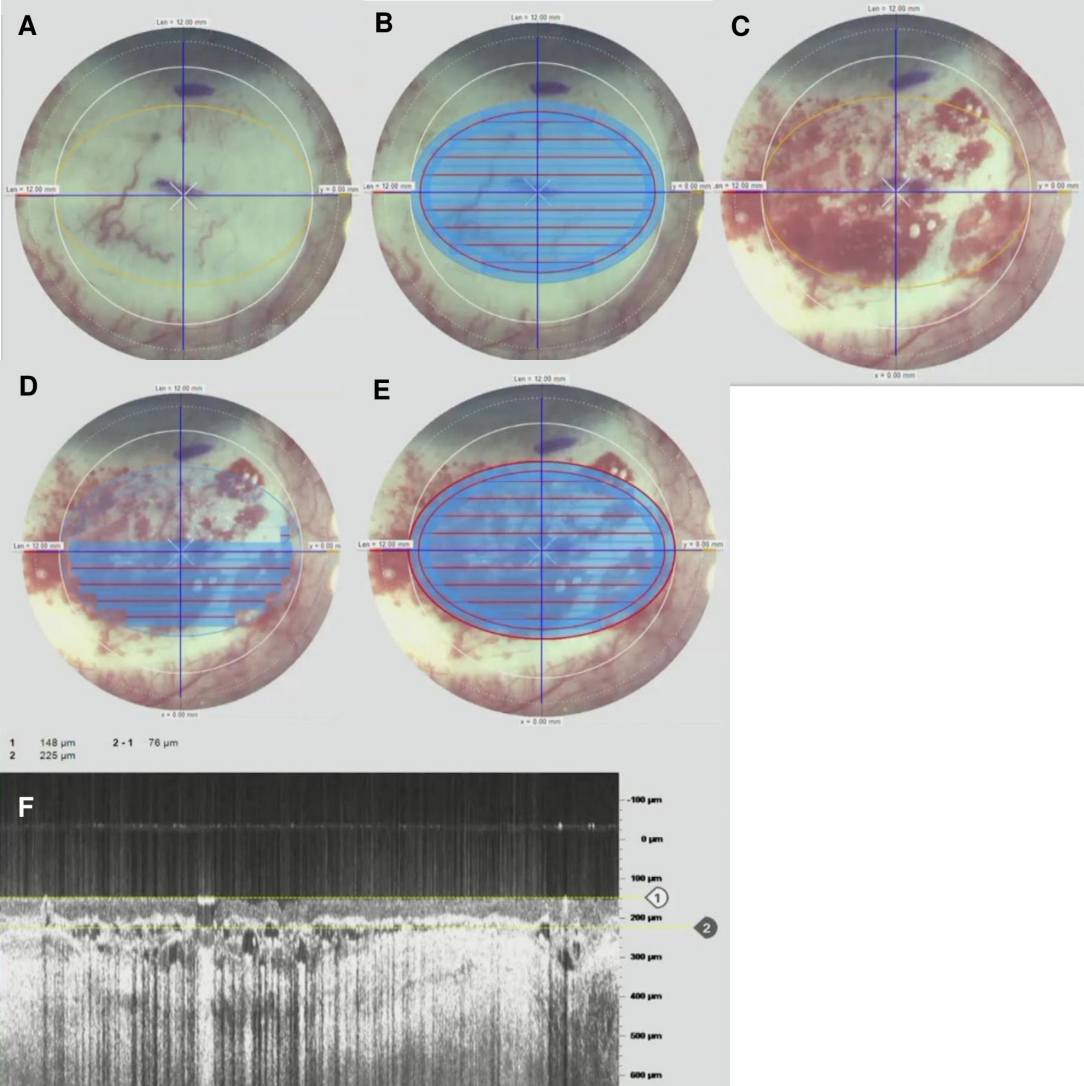
**(A)** Placement of the crosshair of the Z8 visualisation screen on the conjunctival mark made for the intended CAG. **(B)** Lamellar cut of the CAG before displacement occurred. **(C)** Movement of the eye occurred right before the vertical rim cut; the procedure was stopped and FSL was re-applanated on the conjunctiva before restarting CAG dissection. **(D)** Ongoing vertical FSL dissection of CAG. **(E)** CAG dissection completed successfully. **(F)** Central CAG thickness measurement on the built-in optical coherence tomography (OCT) showing central CAG thickness of 76 μm.

Despite FSL micro-movements, the majority of our patients (65.7%) had relatively easy peels. This is likely due to the loose anatomy of the conjunctiva since cavitation bubbles formed during dissection still move into the plane of least resistance and cause little impact on the plane of resection. In a recent study by Liu et al on the 1-year results of a clinical trial of FLAPS, a similar complication rate of 5% was reported [16]. In the event of a large vertical displacement before the end of photodisruption, the surgeon could stop the FSL application and re-applanate the laser on the conjunctiva before proceeding to restart FSL dissection. (**Fig 6**) This would greatly minimise the risk of buttonholes or incomplete CAG tears. Besides, we found a low complication rate of 6% in our study (1 case of buttonhole and 2 of CAG tears) out of 35 eyes. As anticipated, these 3 complicated cases consisted of grade 3 and 4 peels, had above average peel times, and greater vertical FSL displacements. Despite so, these grafts were deemed of adequate quality to be used clinically.

Vertical, horizontal, or absolute FSL displacements also did not influence final CAG thickness, supporting the finding that accurate and reliable preparation of ultra-thin CAGs can be achieved. This was evident when we compared eyes with less than 100 μm to those with more than 100 μm absolute FSL displacements and found no significant differences in terms of final graft thickness or deviation from the originally planned graft thickness. This was also the case amongst eyes with grade 1 to 4 peels or varying duration of peels. A caveat to this is that the thickness measurements are calculated in the central 4 mm zone of the graft. However, we previously reported that in porcine eyes, the FSL produced planar ultra-thin grafts [15]. Liu et al subsequently showed that these FSL grafts of uniform thickness and minimal Tenon’s tissue had no recurrence and graft retraction at the 1-year follow up [16]. We do acknowledge that study sample size calculation of 35 was based on our primary outcome of CAG peel grade and duration and not on other secondary outcomes such as CAG thickness or deviation from intended thickness.

Age and race did not have any correlation with the duration and ease of CAG peel or final CAG thickness. The median age of our study cohort was 65 years (IQR 55.5 to 72 years). There is evidence of age-related thinning of bulbar conjunctiva [19, 20]. However, we did not find that age was correlated with CAG peel quality and duration in our study. Of the 2 eyes with buttonhole and graft tears during the CAG peel, they were 56 and 68 years old respectively. Older patients, i.e. 65 years old, are known to have a thinner and more atrophic Tenon’s [21]. Hence, there was possibly less laser interference and a good quality peel in most of our patients despite minor FSL displacements because of the age of our patients.

The majority of our study cohort were Chinese (75%), with Malays and Indians consisting of the minority. There is some evidence of anatomical differences in Tenon’s capsule layers between races which could potentially affect the quality and duration of CAG peel. Darker skinned-races have multiple layers of Tenon’s capsule due to more cell layers of more compact stratum corneum layers, as observed in African Caribbean eyes compared to that of the general population [22, 23]. A case series of glaucoma filtration surgery failure rates among African Caribbean populations showed greater wound healing responses to surgical trauma with the formation of episcleral and Tenon’s capsule fibrotic tissues [24]. No significant impact of race was noted on CAG peel quality and duration in our study, perhaps attributed to the FSL being programmed to dissect above the Tenon’s layer. The 2 eyes which had complications of buttonhole or incomplete cuts were both ethnically Chinese. However, as mentioned earlier, our sample size calculation of 35 was based on our primary outcome of CAG parameters and not on other secondary outcomes such as CAG thickness or race. Our study would be underpowered to detect any small but significant effects of race on CAG peel quality and duration. Racial distribution in our study was also skewed towards the majority (75%) being Chinese and 10% from darker-skinned races, leading to false negatives in our results.

We acknowledge the limitations in this prospective study with a relatively small number of cases, especially of those falling in Grade 3 or 4 CAG peel categories, that might be underpowered to detect any significant differences of the other variables between groups such as age, race or CAG thickness and deviation from intended thickness, albeit statistically powered to detect a correlation between absolute FSL displacement and CAG parameters as a primary outcome. Also, majority of our eyes undergoing FLAPS fall into Grade 1 and 2 peel categories (75% in our study), with a significantly high success rate of the laser in achieving good CAG peels (based on Kruskal-Wallis test with p<0.001 for distribution of different peel grades). Despite increasing study numbers, we would likely still have difficulty recruiting more grade 3 and 4 peel eyes. As FLAPS is a relatively new technique, this treatment option was only available for consultant surgeons in our centre, which may explain the small FSL displacements recorded. In the future, evaluation of displacements between more surgeons as well as between surgeons compared to surgeons-in-training will be needed. Nonetheless, this is the first study to our knowledge, evaluating the correlation between FSL displacements during CAG creation with the thickness and quality of grafts. We used standardised measurements of CAG peel duration and vertical/horizontal/absolute FSL displacements during the CAG dissection for this study, allowing us to better understand how to maintain the quality of CAG dissection. Although it is feasible to site the FSL-dissected CAG at in the inferior bulbar conjunctiva, it is more challenging to obtain a large CAG from the inferior position.

In conclusion, we have demonstrated that vertical micro-movements of eye during suction-free CAG preparation were associated with worse quality and slower CAG peels, stressing the importance of maintaining vertical globe stability during the procedure. We can be reassured however that despite micro-movements, the quality and duration of the CAG peel or final CAG thickness were not affected. Age and race were not associated with the quality of CAG peel, duration of peel, or final CAG thickness. The main advantage of FLAPS would be the consistent, fast ultra-thin CAG dissection that may require a longer learning curve for surgeons to be able to achieve using a manual technique [25]. This is supported by good surgical outcomes, minimal discomfort, low recurrence, and complication rates [16]. Suction-free FLAPS is a safe procedure and can be considered as an alternative technique for efficient and consistent CAG preparation, increasing the utility of the FSL.
